# Supplementary material for: Guidelines’ recommendations for the treatment-resistant depression: A systematic review of their quality
Source: PLoS One. 2023 Feb 6;18(2):e0281501. doi: 10.1371/journal.pone.0281501 (PMC9901785; doi:10.1371/journal.pone.0281501)
Supplement: S1 File — The search strategy used in the PubMed (Medline), Embase, Cochrane Library, PsycINFO, and BVS databases. (DOCX) [file pone.0281501.s001.docx]

Search strategy used in the PubMed (Medline), Embase, Cochrane Library, PsycINFO, and BVS databases

**Medline (via PubMed)**

(((((("Guideline" [Publication Type] OR "CPGs as Topic"[Mesh] OR "Practice Guideline" [Publication Type] OR "Health Planning CPGs"[Mesh]) OR "Clinical Protocols"[Mesh])) OR ("Consensus Development Conference, NIH" [Publication Type] OR "Consensus Development Conference" [Publication Type] OR "Consensus"[Mesh]))) OR "Standard of Care"[Mesh])) "Guideline" [Publication Type] OR "CPGs as Topic"[Mesh] OR "Practice Guideline" [Publication Type] OR "Health Planning CPGs"[Mesh]) OR "Clinical Protocols"[Mesh])) OR ("Consensus Development Conference, NIH" [Publication Type] OR "Consensus Development Conference" [Publication Type] OR "Consensus"[Mesh]))) OR "Standard of Care"[Mesh])))))) AND (("Depressive Disorder"[Mesh] OR "Depressive Disorder, Major"[Mesh] OR Depressive Disorders OR Disorder, Depressive OR Disorders, Depressive OR Neurosis, Depressive OR Depressive Neuroses OR Depressive Neurosis OR Neuroses, Depressive OR Depression, Endogenous OR Depressions, Endogenous OR Endogenous Depression OR Endogenous Depressions OR Depressive Syndrome OR Depressive Syndromes OR Syndrome, Depressive OR Syndromes, Depressive OR Depression, Neurotic OR Depressions, Neurotic OR Neurotic Depression OR Neurotic Depressions OR Melancholia OR Melancholias OR Unipolar Depression OR Depression, Unipolar OR Depressions, Unipolar OR Unipolar Depressions))

**Embase:**#1 'practice guideline'/mj OR 'consensus development'/exp/mj OR 'clinical protocol'/mj
#2 'depression'/exp
#3 #1 AND #2

**Cochrane Library**

# 1—MeSH descriptor: [Guideline] explode all trees

# 1—MeSH descriptor: [Consensus] explode all trees

# 1—MeSH descriptor: [Clinical Protocols] explode all trees

# 1—#1 OR #2 OR #3

# 1—MeSH descriptor: [Depression] explode all trees

**PsychINFO**

((Any Field: (depression))) AND ((Any Field: (guideline)) OR (Any Field: (consensus)) OR (Any Field: ("clinical protocol"))) AND Year: 2011 To 9999

((Any Field: (depression))) AND ((Any Field: (guideline)) OR (Any Field: (consensus)) OR (Any Field: ("clinical protocol"))) AND Year: 2011 To 9999

**BVS**

((guideline) OR (consensus) OR (clinical protocol)) AND (depression) AND (db:("LILACS" OR "IBECS" OR "WHOLIS" OR "BDENF" OR "BINACIS" OR "INDEXPSI" OR "BIGG" OR "BBO" OR "CUMED")) AND (year_cluster:[2011 TO 2021])
