# Supplementary material for: Guidelines’ recommendations for the treatment-resistant depression: A systematic review of their quality
Source: PLoS One. 2023 Feb 6;18(2):e0281501. doi: 10.1371/journal.pone.0281501 (PMC9901785; doi:10.1371/journal.pone.0281501)
Supplement: S1 Fig — (DOCX) [file pone.0281501.s002.docx]

Figure 1. Flowchart of CPG selection.

**Identification of studies via other methods**

**Identification of studies via databases and registers**

Records identified from:

Websites and Organisations (n = 292)

Records removed *before screening*:

Duplicate records removed

(n = 419)

Records identified from:

Databases (n = 5063)

798 Medline

157 Cochrane Library

1511 Embase

2287 Psycoinfo

310 BVS

**Identification**

Records screened

(n = 4644)

Records excluded

(n = 4468)

Reports not retrieved

(n = 0)

Reports sought for retrieval

(n = 80)

Reports sought for retrieval

(n = 176)

Reports not retrieved

(n = 2)

**Screening**

Reports excluded

70 were not CPGs

18 were duplicates

4 were outdated

9 did not include pharmacological treatment

1 focused on psychotherapy

7 focused on specific populations

10 subject matter

7 specific drugs

Reports excluded:

5 were not CPGs

10 were duplicates

24 did not include pharmacological treatment

1 focused on psychotherapy

16 focused on specific populations

9 subject matter

Reports assessed for eligibility

(n = 80)

Reports assessed for eligibility

(n = 174)

Guidelines included in review

(n = 63)

48 documents from the 5 large bases + 15 from specific bases

**Included**

Page MJ, McKenzie JE, Bossuyt PM, et al. The PRISMA 2020 statement: An updated guideline for

reporting systematic reviews. *Br Med J*. 2021;372:n71. doi: 10.1136/bmj.n71. For more information, visit: [http://www.prisma-statement.org/](about:blank).
